# Development, effectiveness and cost-effectiveness of a new out-patient Breathlessness Support Service: study protocol of a phase III fast-track randomised controlled trial

**DOI:** 10.1186/1471-2466-12-58

**Published:** 2012-09-19

**Authors:** Claudia Bausewein, Caroline Jolley, Charles Reilly, Paula Lobo, Jane Kelly, Helene Bellas, Preety Madan, Caty Panell, Elmien Brink, Chiara De Biase, Wei Gao, Caroline Murphy, Paul McCrone, John Moxham, Irene J Higginson

**Affiliations:** 1 King’s College London, Cicely Saunders Institute, Department of Palliative Care, Policy and Rehabilitation, London, UK; 2 Interdisciplinary Centre for Palliative Medicine, University Hospital Munich, Munich, Germany; 3 Respiratory Medicine, King's College Hospital NHS Foundation Trust, London, UK; 4 Palliative Care Team, King's College Hospital NHS Foundation Trust, London, UK; 5 Physiotherapy, King's College Hospital NHS Foundation Trust, London, UK; 6 Occupational Therapy, King's College Hospital NHS Foundation Trust, London, UK; 7 Macmillan Information and Support Centre, Cicely Saunders Institute, London, UK; 8 King's Clinical Trials Unit, Department of Biostatistics, Institute of Psychiatry, King’s College London, London, UK; 9 King's College London, Health Service and Population Research Department, Institute of Psychiatry, London, UK

## Abstract

**Background:**

Breathlessness is a common and distressing symptom affecting many patients with advanced disease both from malignant and non-malignant origin. A combination of pharmacological and non-pharmacological measures is necessary to treat this symptom successfully. Breathlessness services in various compositions aim to provide comprehensive care for patients and their carers by a multiprofessional team but their effectiveness and cost-effectiveness have not yet been proven. The Breathlessness Support Service (BSS) is a newly created multiprofessional and interdisciplinary outpatient service at a large university hospital in South East London. The aim of this study is to develop and evaluate the effectiveness and cost effectiveness of this multidisciplinary out–patient BSS for the palliation of breathlessness, in advanced malignant and non-malignant disease.

**Methods:**

The BSS was modelled based on the results of qualitative and quantitative studies, and systematic literature reviews. A randomised controlled fast track trial (RCT) comprising two groups: 1) intervention (immediate access to BSS in addition to standard care); 2) control group (standard best practice and access to BSS after a waiting time of six weeks). Patients are included if suffering from breathlessness on exertion or at rest due to advanced disease such as cancer, chronic obstructive pulmonary disease (COPD), chronic heart failure (CHF), interstitial lung disease (ILD) or motor neurone disease (MND) that is refractory to maximal optimised medical management. Both quantitative and qualitative outcomes are assessed in face to-face interviews at baseline, after 6 and 12 weeks. The primary outcome is patients' improvement of mastery of breathlessness after six weeks assessed on the Chronic Respiratory Disease Questionnaire (CRQ). Secondary outcomes for patients include breathlessness severity, symptom burden, palliative care needs, service use, and respiratory measures (spirometry). For analyses, the primary outcome, mastery of breathlessness after six weeks, will be analysed using ANCOVA. Selection of covariates will depend on baseline differences between the groups. Analyses of secondary outcomes will include patients’ symptom burden other than breathlessness, physiological measures (lung function, six minute walk distance), and caregiver burden.

**Discussion:**

Breathlessness services aim to meet the needs of patients suffering from this complex and burdensome symptom and their carers. The newly created BSS is different to other current services as it is run in close collaboration of palliative medicine and respiratory medicine to optimise medical care of patients. It also involves professionals from various medical, nursing, physiotherapy, occupational therapy and social work background.

**Trial registration:**

ClinicalTrials.gov (NCT01165034)

## Background

Breathlessness is a common, distressing symptom in advanced malignant and non-malignant disease, causing considerable disability for patients, and anxiety and social isolation for family and carers [1-3]. Breathlessness is particularly prevalent and difficult to manage in the late terminal stages of disease. Studies have shown that 94% of patients with chronic lung disease [4], 78% of those with lung cancer [5] and more than 50% of patients with heart disease [6] suffer from breathlessness in the last year of life. Most studies examining the progression of breathlessness in cancer over time show increased severity of breathlessness towards death [7,8]. The US SUPPORT study reported that around 70% of COPD patients experienced moderate to severe breathlessness in the last six months of life, increasing to more than 80% over the final 3 days [4]. As a result, breathlessness in end-stage disease is responsible for significant healthcare resource usage with up to 60% hospital admission rates in patients presenting with breathlessness to a cancer treatment centre [9].

Once treatment aimed at slowing progression of the underlying disease has been optimised, palliation of breathlessness usually involves a combination of pharmacological (opioids, benzodiazepines, oxygen therapy) and non-pharmacological (rollator devices, fan therapy, breathing control) strategies but one single intervention will probably not help sufficiently with breathlessness [10]. Multidisciplinary rehabilitation programmes, incorporating exercise training and education, have been shown to reduce breathlessness and improve exercise capacity in COPD [11], heart failure [12], and cancer [13]. However, patients with advanced disease are often too ill to attend pulmonary rehabilitation programmes. In the 1990s, Corner et al developed and tested a nursing clinic for lung cancer patients offering breathing control, activity pacing, relaxation techniques, and psychosocial support [14,15]. In a multi-centre RCT, the intervention group had significantly less breathlessness, better performance status, and lower levels of depression compared to best usual care [14]. Based on these experiences, Booth et al developed a multiprofessional Breathlessness Intervention Service (BIS) [16,17] which is currently under evaluation following the MRC framework for complex interventions [18]. The BIS is offering specialist advice to manage breathlessness in patients with disease of any aetiology. The accessibility of breathlessness services is, however, not uniform across the NHS. Difficulties with provision result, in part, from a lack of evidence as to the most effective, and cost-effective, organisational strategies.

We therefore designed a study to develop and evaluate a new multiprofessional and interdisciplinary Breathlessness Support Service (BSS) in Southeast London for people suffering from breathlessness due to advanced disease and their families/carers. We present the protocol of the intervention and the trial to ensure independence of the results and to stimulate criticism and suggestions from the journal readers.

### Aims and objectives

The aim of this study is to develop and evaluate the effectiveness and cost effectiveness of a multidisciplinary out–patient Breathlessness Support Service (BSS) for the palliation of breathlessness, in advanced malignant and non-malignant disease at King’s College Hospital London.

#### Objectives are

1. To assess the effect of the BSS on patients’ mastery of breathlessness and other breathlessness measures.

2. To assess the effect of the BSS on physiological outcome measures.

3. To assess the effect of the BSS on caregiver burden.

4. To compare NHS resource usage and costs in the two study arms.

5. To explore patients’ experiences using the BSS.

## Methods/Design

### Modelling and development of BSS

The BSS was designed following the Medical Research Council (MRC) Guidance for the development and evaluation of complex interventions [18,19]. This approach has been used to develop and evaluate a range of services and interventions in palliative care [16,20]. This phase III study builds on already completed pre-clinical/ theoretical (0), modelling (I) and exploratory (II) phases which are described below.

#### Phase 0: preclinical/ theoretical phase

The theoretical basis of the BSS was established by reviewing published literature on the impact of breathlessness on healthcare usage in palliative care, and evaluations of breathlessness services that are already in place. The magnitude of the reduction in healthcare resource usage by existing breathlessness advisory services [21,22] and corroborative evidence from related rehabilitative and educational programmes [12,13] was also sought. Evidence for the efficacy of pharmacological and non-pharmacological palliation of breathlessness was reviewed [13,23-25]. This allowed specification of the active ingredients of the intervention, in terms of both the personnel mix and management strategies that would be offered in the service.

#### Phase I: modelling

We conducted qualitative interviews with over 80 patients with COPD, motor neuron disease, and cancer, and their families and caregivers both in the clinical setting (rehabilitation classes, out-patient clinics)[26-29], and informally in patient groups such as the British Lung Foundation Breathe Easy groups. Patients’, carers’ and healthcare professionals’ views on factors precipitating emergency health resource use, such as Accident and Emergency visits were also sought. The issues raised have focussed our intervention and include: need to provide educational and training material, need to have a multi-professional approach, need to include an optional home visit assessment, the need to have a plan of how to deal with crises and need to include support for caregivers. These patient interviews have helped to define our target group, to better understand the relationships between the proposed components of the breathlessness service, and the potential for improved cost-effectiveness of the service compared to best usual medical care. In addition, we have consulted widely within the King’s College Hospital Trust.

### Setting

The BSS is provided as an outpatient clinic based in the Cicely Saunders Institute at King’s College Hospital in Southeast London. The hospital serves an estimated population of 3.5 million people, and has approximately 1400 hospital admissions for breathlessness (=3700 bed days) per year. The area has a network of palliative care services including hospices, community services and hospital support teams, co-ordinated through the South London Palliative Care Network and other regionally based networks.

### Study design of phase III RCT

A randomised controlled fast track trial (RCT) comprising two groups: 1) intervention (immediate access to BSS in addition to standard care); 2) control group (standard best practice and access to BSS after a waiting time of six weeks). Consenting participants are randomly allocated to one of the groups after baseline assessment. The fast track design gives all patients the opportunity to access the service. Patients will continue to have the same access to specialist medical and palliative care services as was available to them prior to entry into the trial. This approach is based on a similar study design developed by Higginson et al, to evaluate the effectiveness of a new palliative care and neurology service for patients severely affected by Multiple Sclerosis [20].

### Inclusion criteria

Patients suffering from breathlessness on exertion or at rest due to advanced disease such as cancer, chronic obstructive pulmonary disease (COPD), chronic heart failure (CHF), interstitial lung disease (ILD) or motor neurone disease (MND). The underlying disease should be optimally medically managed. Patients must be able to engage with short term physiotherapy. If patients are suffering from acute exacerbations, they are put on a waiting list for two weeks and are then entered into the trial.

### Exclusion criteria

Patients with breathlessness of unknown cause, and patients with a primary diagnosis of chronic hyperventilation syndrome are excluded from study participation. Patients are also excluded if they are too ill to come to the clinic, and are unable to provide informed consent either due to cognitive problems or to the severity of their illness.

### Intervention

The BSS is a multi-professional and interdisciplinary service provided by two doctors, a clinical nurse specialist for lung cancer, a respiratory physiotherapist, an occupational therapist, and a social worker. Doctors come from a palliative medicine and a respiratory medicine background.

Out-patient clinics take place once a week. Patients are invited to come twice to the clinic within 4 weeks and will also receive a home visit. Patients see 1-2 health professionals per visit. Each patient is discussed by the respiratory and palliative care professionals before the patient is seen. The patient is reviewed by the respiratory doctor first, who then hands over to the palliative care professional after seeing the patient. Details of the service are given in Table 1.

**Table 1:** Details of Breathlessness Support Service

**Time**	**Type of contact with clinic**	**Content of meeting**
Week 1	First outpatient clinic visit	**Contact with respiratory medicine**
		· explore the symptom of breathlessness and its triggers
		· establish underlying cause of breathlessness
		· optimise disease-orientated management
		· review of previous investigations
		**Contact with palliative medicine**
		· experience of breathlessness
		· development of crises plan
		· burden on patient & family
		· symptom burden (other than breathlessness)
		· psychosocial & spiritual issues
		· introduction of non-pharmacological measures such as rollator and hand-held fan
Week 2 – 3 Week 2 - 3	Home visit Telephone call	Based on the patients’ needs as assessed during home visit:
		**Physiotherapy input**
		· review of the positions of breathlessness
		· provision of a walking aid
		· breathing control techniques and anxiety-panic cycle
		· management of exacerbations in COPD
		· home programme of exercise (DVD, personalised sheet)
		· cough minimisation techniques
		· pacing and fatigue management
		· sputum clearance techniques
		· ambulatory oxygen assessments
		· referral to pulmonary rehabilitation
		**Occupational therapy input**
		· assessment of ADL (mobility / transfers, self care and domestic ADL)
		· assessment for aids and minor adoptions and referral for provision of equipment
		· wheelchair prescription
		· education on planning, pacing and energy conservation techniques to patients and carers
		· referral to other community services (local / out of area), as appropriate
		· assessment of need for social support and liaison with the BSS social worker, as appropriate
		· liaison with the BSS team regarding interventions and feedback
		**Social worker input**
		patient and or carer assessment including understanding of disease and symptoms & information needs and coping strategies
Week 4 - 5	second clinic visit	**Contact with palliative medicine**
		· re-evaluation of breathlessness and other symptoms
		· checking of interventions
		· referral to medical and/or palliative care services if appropriate
		· discharge from service

At the first meeting, patients receive an information pack with five fact sheets on breathlessness, a breathlessness mantra, a relaxation CD, a hand-held fan, and information about cooling the face, e.g. with a small spray bottle spraying fine mist. The fact sheets were developed by Sara Booth et al in Addenbrooke’s Hospital, Cambridge (http://www.cuh.org.uk/addenbrookes/services/clinical/breathlessness_intervention_service/patient_information_leaflets.html), containing information on breathlessness: information and commonly asked questions; managing breathlessness; handheld fan; positions to ease breathlessness; a distraction technique: using the five senses. If necessary, patients are also provided with information more tailored to their specific needs.

The patients receive a letter after the clinic visits with copies to the GPs and the referrers summarizing what has been discussed and recommended.

After the first appointment in the clinic and initial screening of patients’ needs, the physiotherapist (PT) in liaison with the occupational therapist (OT) provides support within three weeks of patient’s first attendance at the clinic. After arranging a suitable time by telephone either PT or OT visits the patients at home or a joint home visit is considered, as necessary. OT and PT aim to facilitate independence, safety and quality in daily activities, using a client centred approach. The visits aim to identify and modify domestic factors that may be negatively impacting on breathlessness and create an individualised functional programme. The therapists also refer to other rehabilitation if appropriate.

Following their initial clinic visit each patient and/or carer will be contacted via telephone by the BSS social worker and assessed to see whether subsequent social work involvement is indicated or referral to other supportive services are required.

### Control group

Patients randomised to the control group continue with optimal medical management as prescribed by their healthcare provider with the same access to generalist (GP, district nurse) and specialist medical (respiratory, cardiology, neurology) and palliative care services (community, hospital, hospice) as was available to them prior to entry into the trial. After the final assessment (6-7 weeks), patients will be entered into the intervention arm of the study (the BSS).

### Recruitment procedure

Participants are recruited to the study by referral from respiratory medicine, pulmonary rehabilitation, cardiology, palliative care services, community services, and GPs. The referring clinicians gain permission from patients for their details to be passed to the study team. The clinical research fellow (CRF), using a standard protocol, screens referrals and sends a letter giving information about the trial of the new service and an invitation to participate, before telephoning patients several days after receipt of the letter to agree consent. The CRF then arranges an appointment for the baseline assessment with the patient and, ideally, the carer. This first appointment takes place in the patient’s home. At this appointment, written informed consent for participation in the study is obtained from participants.

### Randomisation and blinding

Allocation to the intervention group (IG) or control group is performed by the Clinical Trials Unit at King’s College London, following the baseline interview, independently of the research and clinical team. As the group under study is heterogeneous with regard to some key variables, minimisation was chosen as a method for allocation in order to minimise the imbalance between the treatment groups with respect to baseline characteristics [30]. Factors considered are cancer versus non-cancer, breathlessness severity (NRS >3), presence of an informal caregiver and ethnicity (white versus other). The BSS clinical administrator is informed about the allocation via e-mail and sends appointment letters to patients and arranges subsequent clinic appointments. This allows the CRF and the research nurses to be blind to which study arm patients have been assigned. Patients are asked not to tell the CRF and research nurses to which arm they were allocated.

### Timing of data collection

**Figure 1:**
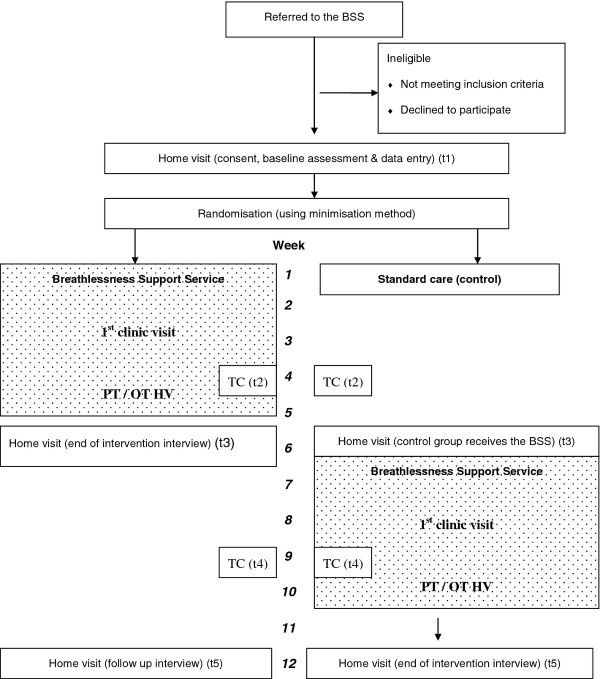
Flow diagram of patients through the study.

Data is collected simultaneously in both groups, as summarised in Figure 1. Baseline data (prior to randomisation) (t1) is collected in each patient’s home by the CRF. This includes all questionnaires mentioned below and physiological tests using a portable spirometer (Vitalograph Gold Standard®,Vitalograph® Buckingham, UK). Three to four weeks after the baseline assessment all patients receive a telephone call from the CRF, during which each patient completes the mastery section of the CRQ (t2). Baseline assessment measures are repeated in the patients home six to seven weeks after entry to the study (t3). These data will be used for analysis of primary outcomes.

Subsequently, patients complete a second telephone questionnaire as previously described between week nine and ten of the study (t4). Finally baseline measures are repeated between week 12 and 13 in all patients (t5), these data will be used for analysis of secondary outcomes.

The timing of interventions and data collection has been designed to allow for short disease trajectories in patients with cancer and minimise patient burden, whilst allowing time for interventions to have the desired effect. Four weeks is considered to be the minimum length of pulmonary rehabilitation programmes that give a clinically significant benefit [31]. Measures for each time points are displayed in Table 2.

**Table 2:** Measures for each time point

	**Baseline**	**4 weeks (telephone)**	**6 weeks**	**8 weeks (telephone)**	**12 weeks**
Chronic Respiratory Disease Questionnaire (CRQ)	x	x	x	x	x
NRS breathlessness	x		x		x
Dyspnoea 12	x		x		x
Palliative Care Outcome Scale (POS)	x		x		x
POS Symptom Scale (POS-S)	x		x		x
London Chest Activity of Daily Living Scale (LCADL)	x		x		x
Hospital Anxiety and Depression Scale (HADS)	x		x		x
EuroQol (EQ-5D)	x		x		x
Zarit Burden Inventory	x		x		x
Client Service Receipt Inventory (CSRI)	x		x		x
Spirometry	x		x		x

### Outcome measures

Both quantitative and qualitative outcomes are assessed. Baseline data includes age, sex, clinical diagnosis, height, weight and smoking history. Data are collected from participants by face-to-face interviews at two time-points and telephone interviews.

#### Primary outcome measure

The primary outcome is improvement of mastery of breathlessness after 6 weeks, as assessed by a change in the score in the Mastery domain of the Chronic Respiratory Disease Questionnaire (CRQ) [32]. Questions within this domain relate to breathlessness. The CRQ is a multidimensional tool, and is one of the most widely used measures of quality of life (QOL) in chronic respiratory disease. The CRQ is an interview administered questionnaire, but a self-administered version maintains validity and responsiveness [33].

#### Secondary outcome measure

##### Breathlessness

A Numerical Rating Scale (NRS) [34] is used as secondary outcome measure to assess the intensity of the sensation of breathlessness on average, at worst, at rest and on exertion over the last 24 hours. The NRS correlates highly with the visual analogue dyspnoea scale [34], and has the advantage of being able to be used during the telephone assessments. In addition, we included the Dyspnoea 12, a newly validated measure which provides a global score of breathlessness severity that incorporates both “physical” and “affective” aspects, and can measure dyspnoea in a variety of diseases [35].

##### Symptom-related QOL

The Palliative care Outcome Scale (POS) and POS-symptoms are used to assess and quantify palliative care symptoms in addition to breathlessness [36]. The London Chest Activity of Daily Living Scale (LCADL) is used to assess the impact of the service on activities of daily living. The LCADL has been used as an outcome measure in COPD, and has been shown to be valid, reliable and responsive to change [37]. The Hospital Anxiety and Depression Score is used to screen patients for anxiety and depression [38], and the EQ-5D [39] is used as a generic health-related QOL measure. EQ-5D is a standardised instrument for use as a measure of health outcome and is especially suited to cost-effectiveness analyses as it can be used to generate quality-adjusted life years (QALYs). It is applicable to a wide range of health conditions and treatments, and provides a simple descriptive profile and a single index value for health status. Carer burden is assessed using the Zarit Burden Inventory [40].

##### Service use

Contacts with the BSS are recorded centrally. The Client Service Receipt Inventory (CSRI) is used to record other service use in the three-month period prior to baseline assessment and then for each follow-up period. The CSRI has been used in over 200 studies to assess costs and takes approximately 20 minutes to complete. Services included will be health care (primary and secondary), medication, social care and informal care from family/friends. The CSRI has not previously been used specifically in a breathless population, therefore we adapted the CSRI for this study. Lost work time for patients and carers will be recorded.

##### Physiological measures

These include spirometry (forced expiratory volume in 1s [FEV_1_, slow vital capacity [VC], peak expiratory flow rate [PEF]), performed in accordance with the American Thoracic Society guidelines [41]. The highest value for FEV_1_, VC and PEF from three reproducible efforts will be recorded and compared to predicted normal values [41]. Pulse oximetry is measured at rest and during the six minute walk test [42] in all participants.

When attending the clinic, patients are asked to perform a six minute walk test as a field test of exercise capacity. The distance walked (6 minute walk distance, [6MWD]) has demonstrated strong correlation with peak rate of oxygen uptake, maximum work rate [43] and prognosis in patients with lung cancer [44] and chronic respiratory disease [45].

##### Patients experience with the BSS

A subsample of patients will be interviewed using semi structured in–depth interviews to understand how the intervention might work and what positive and negative aspects patients experienced. Patients will be identified using a sampling frame (see Table 3). The interviews will follow a topic guide which includes questions on patients’ experience and views regarding service provision, location of the service, help and information received, and impact of service. The interviewer will have had no previous contact with the patient during the BSS contact. Interviews will take place at the end of the 12 weeks period when patients have attended the BSS.

**Table 3:** Sampling frame for qualitative interviews

	**Cancer**	**Non-cancer (COPD/ CHF/ILD etc.)**
Severity of breathlessness		
NRS < 3	4 – 6	4 – 6
NRS ≥ 3	4 – 6	4 – 6
Sex		
male	4 – 6	4 – 6
female	4 – 6	4 – 6
Age		
< 50 years	2 – 3	2 – 3
50 – 65 years	2 – 3	2 – 3
≥ 65 years	2 – 3	2 – 3
Caregiver situation		
living with caregiver	4 – 6	4 – 6
no caregiver	4 – 6	4 – 6
Intervention		
Fast-track	4 – 6	4 – 6
Waiting list	4 – 6	4 – 6
**Total**	**10**	**10**

### Sample size calculation

There are limited data to provide estimates for a sample size calculation and this study will inform calculations for the future. Based on the primary outcome, the mastery domain on the CRQ [32]to detect a mean difference of 0.70 on this 7 point scale with a standard deviation of 1 [46], and at 5% significance level, power of 80% we would require at least 33 patients in each group. Due to the advanced stage of illness in the patients we estimate an attrition rate of 40% which means that we would aim to recruit at least 110 patients, which over 12 months, would be 9-10 patients per month. With over 1,000 admissions for breathlessness per year, this should be achieved.

### Analysis

First, we aim to understand missing data in the study and proportions and reasons for drop out. Second, we will compare baseline data between the intervention and the control group using parametric and non-parametric tests as appropriate. Third, the primary outcome, mastery of breathlessness after six weeks, will be analysed using a t-test if there are no differences between groups or ANCOVA with selection of co-variates depending on baseline differences between the groups. Analyses of secondary outcomes will include patients’ symptom burden other than breathlessness, physiological measures, and caregiver burden.

Dealing with missing data will include various imputation methods. The alpha level for all statistical analyses will be set at two-sided 0.05. All analyses will be performed using SPSS software.

Qualitative interviews will be tape-recorded, transcribed verbatim and analysed using content analysis. NVivo is used to facilitate qualitative analyses.

### Economic evaluation

The service use data recorded with the CSRI will be combined with appropriate unit cost information (for example the annual compendium produced by the Personal Social Services Research Unit at the University of Kent). The costs of the BSS itself will be estimated using local accounting data and information on activity rates. Informal care costs will be estimated using the unit cost of a homecare worker. Lost employment will be costed using average wage rates. Cost data are generally skewed and therefore to make comparisons between the groups bootstrapping methods will be used. Cost-effectiveness will be assessed by combining the data on costs with the data on the mastery domain of the CRQ. Cost-utility estimates will be made using QALYs generated from the EQ-5D. If costs are higher and outcomes better for the BSS then incremental cost-effectiveness ratios will be generated which will indicate the extra cost incurred to achieve an extra unit of outcome. Uncertainty around cost-effectiveness and cost-utility estimates will be explored by resampling from the dataset using bootstrapping, calculating cost and outcome differences for each resample and plotting these on cost-effectiveness planes. Cost-effectiveness acceptability curves will be used to show the probability that the BSS is more cost-effective than usual care for a range of values placed on a unit improvement in outcome.

### Project advisory group

A multidisciplinary project advisory group (PAG) has been established for the duration of the study. The PAG includes clinicians and researchers, and others working with breathless patients. The specialities of respiratory medicine and palliative medicine are represented on the PAG, as are local services that linked with the new breathlessness support service. A patient and carer representative are also PAG members. The PAG meets regularly and also make contact by e-mail and telephone with the project team.

### Ethics approval

Ethics approval for the study was gained from the King’s College Hospital ethics committee (Ref. 10/H0808/17). The study meets the requirements of the local Research Governance Framework. The study protocol is registered with ClinicalTrials.gov (NCT01165034).

## Discussion

The complexity of breathlessness, the needs of patients with advanced disease living with this distressing symptom, and the burden of their carers demand a combination of various pharmacological and non-pharmacological interventions. To provide these, various types of breathlessness services have been developed over the last years but their effectiveness and especially cost-effectiveness remains to be shown.

The BSS introduced here was developed following the MRC framework for complex interventions. Building on previous own research [13,26-28,47] and experiences from colleagues in Cambridge [16,17,48] we modelled our service to fit the needs of an ethnically diverse population in South East London. Although there are some similarities to the BIS in Cambridge such as multi-professional support, service for cancer and non-cancer patients, and short-term intervention with consecutive discharge of patients, there are also some pronounced differences. We recognised the need to introduce respiratory expertise in such a service and therefore run, for the first time, an interdisciplinary breathlessness service with colleagues from palliative medicine and respiratory medicine. This allows us to provide special expertise to optimise treatment of the underlying disease but also to include physiological measures in the assessment of patients and the evaluation of the service such as lung function and exercise capacity to better describe the sample and to understand the potential effects of the interventions provided. The composition of professionals being involved in the service has been extended in comparison to other services and includes the expertise of a nurse, an occupational therapist and a social worker. In contrast to the Cambridge Service with provision of the BIS mainly in patients’ homes [49] is the predominant provision of the BSS as an outpatient clinic with one home visit by the physiotherapist or the occupational therapist between two clinic visits.

## Competing interests

The authors declare that they have no competing interests.

## Authors' contributions

IJH, CB, CJ and JM conceived the idea for the study and secured funding. IJH, CB, CJ, CR, CM, JM, GW and PMC set up the study. IJH, JM, CJ, PL, JK, CP, HB, PM, EB and CDB provide the service. CR and CP collect the data. CB supervises CR and oversees the study. GW provides statistical and PMC health economy support. All authors have read and approved the final manuscript.

## Pre-publication history

The pre-publication history for this paper can be accessed here:

http://www.biomedcentral.com/1471-2466/12/58/prepub
